# *In vitro* effects of imatinib on CD34^+^ cells of patients with chronic myeloid leukemia in the megakaryocytic crisis phase

**DOI:** 10.3892/ol.2014.1780

**Published:** 2014-01-02

**Authors:** FANKAI MENG, WEN ZENG, LIFANG HUANG, SHUANG QIN, NINGNING MIAO, HANYING SUN, CHUNRUI LI

**Affiliations:** Department of Hematology, Tongji Hospital, Tongji Medical College, Huazhong University of Science and Technology, Wuhan, Hubei 430030, P.R. China

**Keywords:** imatinib, chronic myelocytic leukemia, megakaryocytic blast crisis

## Abstract

Imatinib is a tailored drug for the treatment of chronic myeloid leukemia (CML), and has substantial activity and a favorable safety profile when used as a single agent in patients with CML in myeloid blast crisis. The megakaryocytic blast crisis in CML occurs rarely and carries a poor prognosis. The aim of the present study was to investigate the effects of imatinib on cluster of differentiation (CD)34^+^ cells from patients with CML in the megakaryocytic crisis phase. Bone marrow mononuclear cells (BMNCs) were isolated from patients with CML in the megakaryocytic crisis phase. CD34^+^ cells were selected from BMNCs by positive immunomagnetic column separation. Imatinib significantly induced G_1_ arrest, reduced the phosphorylation of cyclin-dependent kinase 1 and retinoblastoma proteins and inhibited the proliferation of CD34^+^ cells from patients with CML in the megakaryocytic crisis phase. Annexin V/propidium iodide and caspase-3 activity showed that imatinib induced apoptosis. Western blot analysis and protein tyrosine kinase activity assays showed that imatinib inhibited *BCR-ABL* protein tyrosine kinase activity. The *in vitro* data thus markedly indicate a potential clinical application of imatinib for patients with CML in the megakaryocytic crisis phase.

## Introduction

Blast crisis (BC) is the major remaining obstacle in the management of chronic myeloid leukemia (CML) (1). The treatment of CML has been fundamentally altered through the introduction of imatinib, an inhibitor targeted at the *BCR-ABL* tyrosine kinase (2). Imatinib is able to reduce the expression of *BCR-ABL* to extremely low or non-detectable levels in the majority of patients (3). Imatinib is a potent and selective inhibitor of the *BCR-ABL* protein tyrosine kinase. Imatinib specifically causes growth arrest or apoptosis in *BCR-ABL*-positive cells through competitive inhibition at the ATP-binding site of this enzyme, which prevents the phosphorylation of downstream targets (4). Imatinib has a high specificity for *BCR-ABL*, the receptor for platelet-derived growth factor and c-kit tyrosine kinases, with minimal effects in normal cells. In preclinical studies, imatinib showed specific antileukemic activity *in vitro* and *in vivo* against *BCR-ABL*-positive cells, including the eradication of leukemia induced by the injection of cell lines derived from patients with blast-crisis CML (5–7). Imatinib was observed to induce substantial and durable responses in the majority of patients with chronic-phase CML in a clinical phase I study using ascending doses (8). Imatinib also induced hematological responses in 21 out of 38 patients (55%) with CML in myeloid blast crisis (9). Results of a phase II study of CML in myeloid blast crisis confirmed the activity and safety of imatinib in a larger population of patients (10).

Blast crisis as the first presentation of CML accounts for 5–10% of all cases (11). The presence of translocation t(9;22)(q34;q11) or the *BCR-ABL* fusion gene distinguishes between CML blast crisis and acute myeloid leukemia. To the best of our knowledge, a megakaryocytic blast crisis in CML occurs rarely, but carries a very poor prognosis (11–13). Therefore, it was hypothesized that imatinib could be effective for patients with CML in the megakaryocytic crisis phase. CD34^+^ hematopoietic progenitor cells have a higher drug sensitivity than their progenies (14). Using a variety of assays for cell proliferation, cell cycle distribution, apoptosis and protein turnover/activity of *BCR-ABL* tyrosine kinase, the effects of imatinib on CD34^+^ cells from patients with CML in the megakaryocytic crisis phase were tested in the present study.

## Patients and methods

### Patients

Heparin-treated bone marrow (BM) samples were obtained from three patients with CML in the megakaryocytic crisis phase, three patients with CML in the myeloid crisis phase and three patients with acute megakaryocytic leukemia (AMegL). The three patients with CML in the megakaryocytic crisis phase were characterized with megakaryocytic blast crisis as the first presentation of CML. The study design adhered to the principles of the Helsinki Declaration and was approved by the ethics committees of Tongji Hospital (Wuhan, China). Written informed consent was obtained from the patients. The nine patients ranged in age between 32 and 63 years. At the time of investigation, none of the patients had received previous treatment. The diagnosis of CML was established on the basis of the morphological examination, the presence of the Ph chromosome and positive reverse transcription polymerase chain reaction results for *BCR-ABL* fusion transcripts. CML in blast crisis was defined as the presence of ≥30% of blasts in the peripheral blood or BM. The presence of the myeloid phenotype was confirmed by flow cytometry and required myeloperoxidase positivity, the presence of standard myeloid markers and not more than one lymphoid marker (10). AMegL patients had to fulfill the following criteria: The blast population was required to represent >20% of cells in the BM aspirate, according to the World Health Organization classification (15), and to be myeloperoxidase-negative. Immunophenotypes were required when blasts could not be unequivocally classified as megakaryoblasts based on the morphological criteria. The recommendations of the European Group for the Immunological Classification of Acute Leukemias were then applied to validate the diagnosis; the negativity of lymphoid antigens together with either the positivity of two megakaryoblastic markers (CD41, CD42 or CD61) or the expression of one megakaryoblastic marker associated with CD36 positivity (16).

### Isolation and culture of CD34^+^ cells

Ficoll density gradient centrifugation (specific gravity, 1.077) was used to isolate BM mononuclear cells (BMNCs), then positive immunomagnetic column separation (Miltenyi Biotech, Auburn, CA, USA), was used, according to the manufacturer’s instructions, to select CD34^+^ cells from the BMNCs. The purity of the CD34^+^ cells ranged between 88 and 96% and the viability was >96%, as determined by as determined by flow cytometry (EPICS XL; Beckman Coulter, Miami, FL, USA) and a trypan blue exclusion assay (Sigma, St. Louis, MO, USA), respectively. The cells were cryopreserved in 10% dimethylsulfoxide (Sigma) and 50% fetal bovine serum (Life Technologies Corp., Grand Island, NY, USA) by initial freezing for 24 h at −70°C, followed by storage in a −150°C freezer. Cryopreserved cells were used in the imatinib studies.

CD34^+^ cells from the freezer were cultured in multiwell tissue-culture plates in serum-free medium (StemPro, Life Technologies Corp.), supplemented with growth factors (200 ng/l granulocyte macrophage-colony-stimulating factor, 1 μg/l granulocyte colony-stimulating factor, 50 ng/l leukemia inhibitory factor, 200 ng/l stem cell factor, 200 ng/l macrophage inflammatory protein-1α and 1 μg/l interleukin-6; PeproTech, London, UK) (17).

### Cell lines

The *BCR-ABL*-positive K562 cell line was cultured in RPMI-1640, supplemented with 10% fetal bovine serum in a humidified atmosphere with 5% CO_2_ at 37°C.

### Chemicals

Imatinib was purchased from Novartis (Basel, Switzerland) and dissolved in dimethylsulfoxide to a concentration of 10 mmol/l. To obtain the final concentration, stock solutions were diluted in RPMI-1640 medium.

### Cell proliferation assays

The cell proliferation assay was performed using the MTT-based Cell Growth Determination kit (Sigma), according to the manufacturer’s instructions. K562 and CD34^+^ cells were resuspended in RPMI-1640 medium containing 100 U/ml penicillin, 100 mg/ml streptomycin and 10% fetal bovine serum, and seeded in 96-well microtiter plates with 100 μl per well for quintuplicate wells. The culture medium without cells was used as a blank control. The cells were cultured at 37°C with 5% CO_2_ and 95% humidity for 24 h, followed by incubation in a serum-free medium for another 12 h. Imatinib was added at concentrations of 0.01, 0.05, 0.25, 0.5, 1.0, 2.0 and 10.0 μmol/l, and the incubations were continued for 24, 48 and 72 h. Subsequently, 10 μl MTT (5 mg/ml) was added to each well and the cells were incubated for another 4 h. The plates were centrifuged at a low speed (500 × g) and the supernatants were discarded. Next, 100 μl DMSO was added to each well and the absorbance was measured at 490 nm against the blank control using an ELISA reader.

### DNA content analysis by flow cytometry

The cells (1.0×10^6^/ml) were collected, washed in PBS and fixed in 70% ice-cold ethanol at 4°C for >24 h, followed by incubation with DNase-free RNase (Sigma) for 20 min at 37°C. The cells were stained with propidium iodide (PI, 50 μg/ml) and stored in the dark for 30 min at 4°C. Cell cycle analysis was performed with a FACScan cytometer (FACSsort, Becton Dickinson, San Jose, CA, USA) using Multicycle software (Beckman Coulter).

### Apoptosis assessment by annexin V staining

Following treatment with imatinib for 24 to 72 h, between 1.0×10^5^ and 5.0×10^5^ cells were washed in PBS and resuspended in 200 μl staining solution containing 5 μl fluorescein isothiocyanate-conjugated annexin V and 10 μl 20 μg/ml PI, according to the annexin V staining kit protocol (Clontech, Palo Alto, CA, USA). A total of 5,000 gated events in each sample were analyzed by a Beckman Coulter flow cytometer. The live cells (negative for annexin V and PI), apoptotic cells (annexin V-positive) and necrotic cells (positive for annexin V and PI) were gated according to their fluorescence characteristics. All data were analyzed by Multigraph software (Phoenix Flow Systems, San Diego, CA, USA).

### Caspase-3 activity assay

A ApoAlert™ Caspase-3 Colorimetric Assay kit (Clontech) was used to measure caspase-3 activity, according to the manufacturer’s instructions. A total of 2×10^6^ imatinib-treated cells were lysed. Assays were performed by incubating 100 μg cell lysates in 100 μl reaction buffer [1% Nonidet P-40, 20 mmol/l Tris-HCl (pH 7.5), 137 mmol/l NaCl and 10% glycerol) containing 5 μl caspase-3 substrate, DEVD-pNA, at 37°C for 2 h. A spectrophotometer was then used to measure the absorbance at 405 nm.

### Western blot analysis

A total of 5×10^6^ cells were washed in PBS and lysed using 200 μl radioimmunoprecipitation assay (RIPA) buffer [containing 50 mmol/l Tris (pH 8.0), 150 mmol/l NaCl, 0.1% sodium dodecyl sulfate (SDS), 0.5% deoxycholate, 1% NP-40, 1 mmol/l dithiothreitol (DTT), 1 μmol/l sodium vanadate and 0.2 mmol/l phenylmethyl sulfonic fluoride] following drug treatment for 48 h. The Bradford method (Dc Protein Assay, Bio-Rad, Hercules, CA, USA) was used to determine the protein concentrations. Next, 100 μg total protein was run on 8% SDS-polyacrylamide gels and transferred to polyvinylidene difluoride membranes (Amersham, Buckinghamshire, UK). The membranes were probed with individual antibodies and visualized by an enhanced chemiluminescence system (Pierce, Rockford, IL, USA). The following antibodies were used: Anti-retinoblastoma (anti-Rb), anti-phospho-Rb, anti-cyclin-dependent kinase 1 (anti-CDK1), anti-phospho-CDK1, anti-abl (Santa Cruz Biotechnology, Santa Cruz, CA, USA) and anti-actin (Oncogene, Boston, MA, USA). The secondary antibodies consisted of anti-rabbit peroxidase-conjugated antibody (New England Biolabs, Beverly, MA, USA) and anti-mouse or anti-goat peroxidase-conjugated antibody (Oncogene).

### Protein tyrosine kinase (PTK) activity assay

c-ABL and *BCR-ABL* in samples containing 100 μg total protein were obtained by immunoprecipitation (Santa Cruz Biotechnology). The immunoprecipitates were washed with RIPA buffer and then resuspended in assay buffer [Tris-HCl 50 mmol/l (pH 7.4), MgCl_2_ 40 mmol/l, sodium vanadate 50 mmol/l, DTT 2 mmol/l, MnCl_2_ 1 mmol/l]. Subsequent to centrifugation at 3,000 × g, the precipitated protein was directly assayed for PTK activity, according to the manufacturer’s instructions (SGT410, Chemicon International, Temecula, CA, USA). Each test was performed in triplicate and the results were calibrated with a corresponding phosphopeptide standard curve and control. The absorbance at 450 nm was measured on a spectrophotometer.

### Statistical analysis

Continuous data were expressed as the mean ± SD. Statistical analysis was performed using the SPSS 13.0 software package (SPSS, Inc., Chicago, IL, USA). The Mann-Whitney U test (for continuous variables) or the χ^2^ analysis and Fisher’s exact test (for categorical variables) were carried out to compare the differences between the patient groups. For all analyses, the P-values were two-tailed and P<0.05 was considered to indicate a statistically significant difference.

## Results

### Effect of imatinib on the proliferation of CD34^+^ cells and the K562 cell line

**Figure 1:**
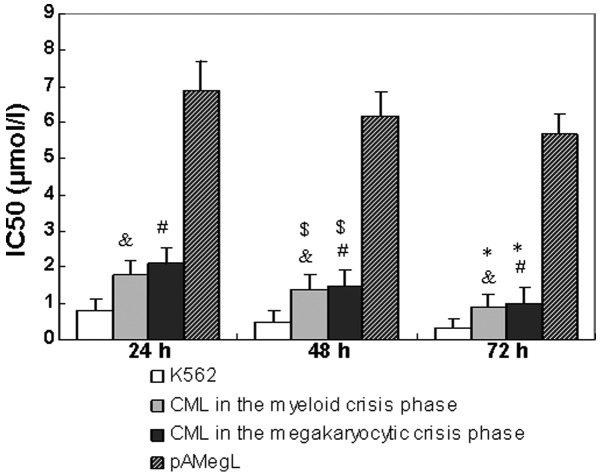
IC_50_ values of CD34^+^ cells and the K562 cell line at 24, 48 and 72 h (n=3, mean ± SD). ^&^P<0.01 (CD34^+^ cells from patients with CML in the myeloid crisis phase vs. K562); ^#^P<0.01 (CD34^+^ cells from patients with CML in the megakaryocytic crisis phase vs. K562); ^$^P<0.05 (48 vs. 24 h); ^*^P<0.05 (72 vs. 48 h). IC_50_, half maximal inhibitory concentration; CD34, cluster of differentiation 34; CML, chronic myeloid leukemia; AMegL, acute megakaryocytic leukemia.

The half maximal inhibitory concentration (IC_50_) values (50% inhibition of proliferation) of imatinib in various cells are shown in Fig. 1. Imatinib produced no measurable effect in the CD34^+^ cells from primary acute megakaryocytic leukemia at therapeutically relevant concentrations (IC_50_, >5 μmol/l). All CML samples were sensitive to imatinib. The mean IC_50_ of imatinib subsequent to 24-, 48- and 72-h incubation periods was 2.1, 1.5 and 1.0 μmol/l for the samples from patients with CML in the megakaryocytic crisis phase, 1.8, 1.4 and 0.9 μmol/l for the samples from patients with CML in the myeloid crisis phase and 0.8, 0.5 and 0.3 μmol/l for the K562 cell line, respectively.

### Effects of imatinib on cell cycle distribution and cell cycle-related protein in CD34^+^ cells

**Figure 2:**
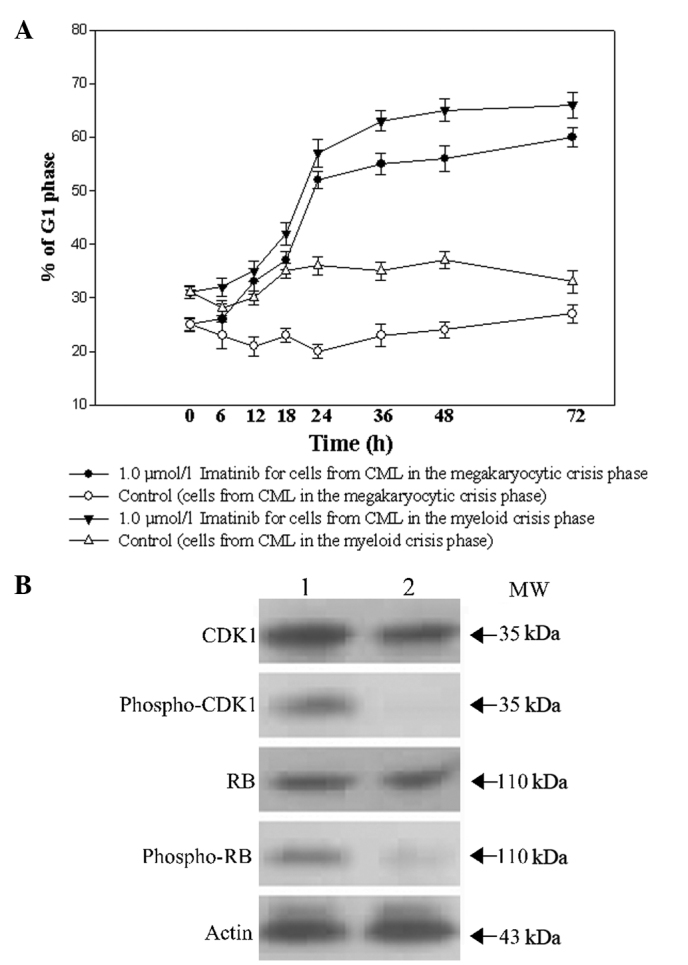
Effects of imatinib on cell cycle distribution and cell cycle-related protein (n=3, mean ± SD). (A) The cell cycle distribution analysis of the CD34^+^ cells from patients with CML in the megakaryocytic crisis phase and in the myeloid crisis phase incubated with 1.0 μmol/l imatinib at different time-points. (B) Western blot analysis of certain cell cycle-related proteins in the CD34^+^ cells from patients with CML in the megakaryocytic crisis phase treated with 1.0 μmol/l imatinib for 48 h. 1, control; 2, 1.0 μmol/l imatinib. CD34, cluster of differentiation 34; CML, chronic myeloid leukemia; CDK1, cyclin-dependent kinase 1; Rb, retinoblastoma; MW, molecular weight.

The effects of imatinib on the cell cycle distribution of the CD34^+^ cells from patients with CML in the myeloid and megakaryocytic crisis phases were evaluated. As shown in Fig. 2A, subsequent to a 24-h exposure to 1.0 μmol/l imatinib, the CD34^+^ cells from patients with CML in the megakaryocytic and myeloid crisis phases began to be arrested at the G_1_ phase. No marked difference between the cells was found. To investigate the mechanism of cell cycle regulation by imatinib, certain associated proteins were examined in CD34^+^ cells from patients with CML in the megakaryocytic crisis phase. As shown in Fig. 2B, no significant changes were observed in total CDK1 and Rb proteins following a 48-h exposure to 1.0 μmol/l imatinib. However, imatinib markedly reduced the phosphorylation of CDK1 and Rb.

### Imatinib-induced apoptosis of CD34^+^ cells

**Figure 3:**
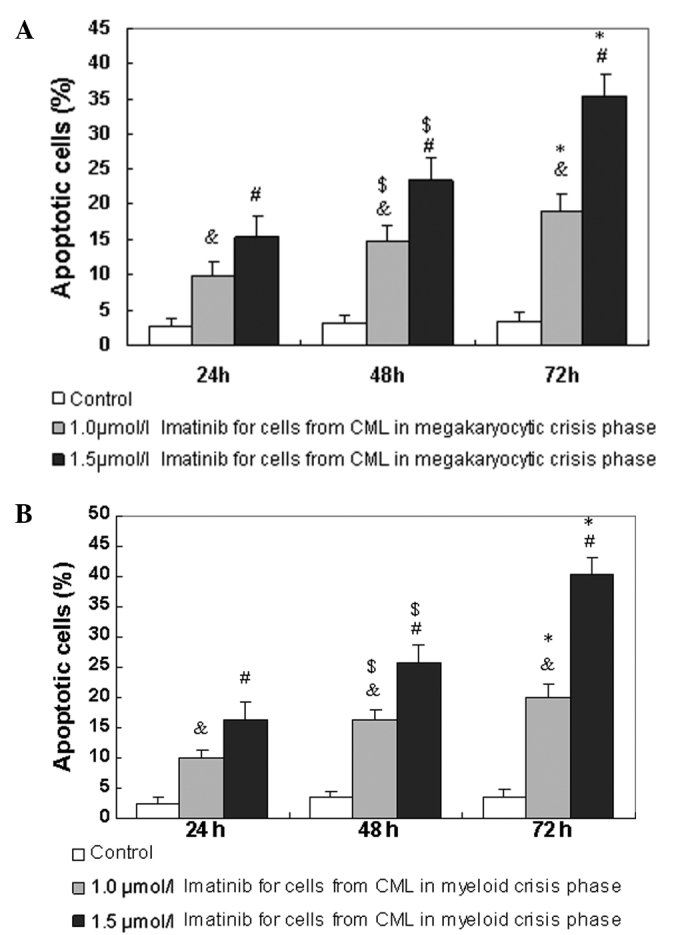
Annexin V-PI assessment of CD34^+^ cells from patients with CML in (A) the megakaryocytic crisis phase and (B) the myeloid crisis phase. Cells were treated with 1.0 or 1.5 μmol/l imatinib for 24 to 72 h and measured for annexin V positivity. (n=3, mean ± SD). ^&^P<0.01 (1.0 μmol/l imatinib vs. control); ^#^P<0.01 (1.5 μmol/l imatinib vs. 1.0 μmol/l imatinib); ^$^P<0.01 (48 vs. 24 h); ^*^P<0.01 (72 vs. 48 h). PI, propidium iodide; CD34, cluster of differentiation 34; CML, chronic myeloid leukemia.

Annexin V is an early indicator of apoptosis. Therefore, the percentages of apoptotic cells were determined using annexin V/PI staining, once CD34^+^ cells from patients with CML in the megakaryocytic and myeloid crisis phases had been exposed to imatinib for 24 to 72 h. Compared with 1.0 μmol/l imatinib for 24 to 72 h, 1.5 μmol/l imatinib resulted in significantly more apoptosis of the CD34^+^ cells from patients with CML in the megakaryocytic crisis phase in a dose- and time-dependent manner (Fig. 3A). The CD34^+^ cells from patients with CML in the myeloid crisis phase also exhibited significant apoptotic changes (Fig. 3B). The apoptotic rate was not notably different between the CD34^+^ cells from patients with CML in the megakaryocytic crisis phase and those from patients with CML in the myeloid crisis phase.

**Figure 4:**
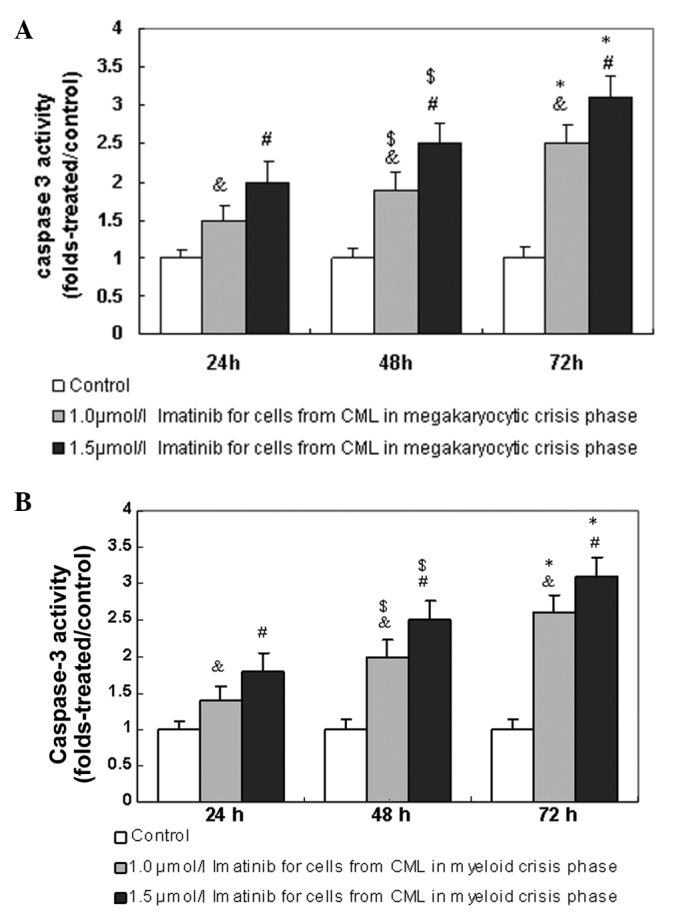
Caspase-3 activity of CD34^+^ cells from patients with CML in (A) the megakaryocytic crisis phase and (B) the myeloid crisis phase following 24 to 72 h of incubation with 1.0 or 1.5 μmol/l imatinib (n=3, mean ± SD). ^&^P<0.01 (1.0 μmol/l imatinib vs. control); ^#^P<0.01 (1.5 μmol/l imatinib vs. 1.0 μmol/l imatinib); ^$^P<0.01 (48 vs. 24 h); ^*^P<0.01 (72 vs. 48 h). CD34, cluster of differentiation 34; CML, chronic myeloid leukemia.

As shown in Fig. 4A, compared with 1.0 μmol/l imatinib for 24 to 72 h, 1.5 μmol/l imatinib resulted in more activated caspase-3 in a higher percentage of CD34^+^ cells from patients with CML in the megakaryocytic crisis phase in a dose- and time-dependent manner. The CD34^+^ cells from patients with CML in the myeloid crisis phase also exhibited more activated caspase-3 (Fig. 4B). The percentage of activated caspase-3 was not notably different between the CD34^+^ cells from patients with CML in the megakaryocytic crisis phase and those from patients with CML in the myeloid crisis phase.

### Effects of imatinib on BCR-ABL protein and its PTK activity

**Figure 5:**
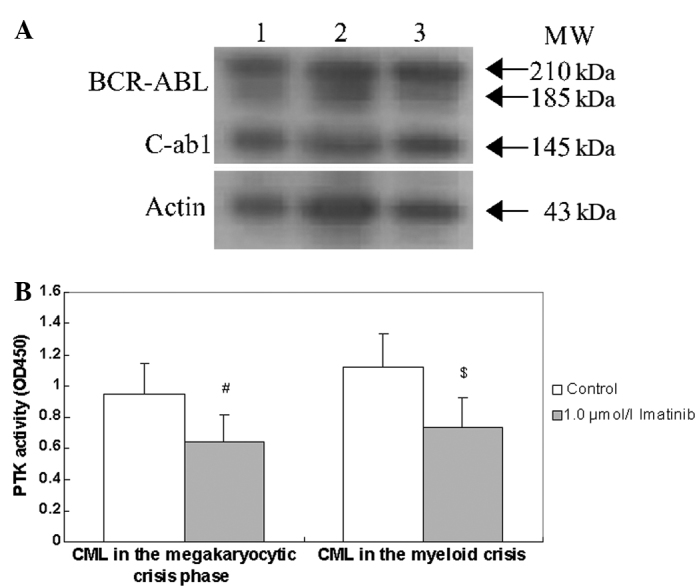
Effects of imatinib on *BCR-ABL* oncoprotein and PTK activity in CD34^+^ cells from patients with CML in the megakaryocytic crisis phase and in the myeloid crisis phase following 48 h of incubation with 1.0 μmol/l imatinib (n=3, mean ± SD). (A) Western blot analysis of BCR-ABL of CD34^+^ cells. 1, Control; 2, CD34^+^ cells from patients with CML in the megakaryocytic crisis phase; 3, CD34^+^ cells from patients with CML in the myeloid crisis phase. (B) PTK activity of CD34^+^ cells. ^#^P<0.05 (CD34^+^ cells from patients with CML in the megakaryocytic crisis phase vs. control). ^$^P<0.01 (CD34^+^ cells from patients with CML in the myeloid crisis phase vs. control). CD34, cluster of differentiation 34; CML, chronic myeloid leukemia; PTK, protein tyrosine kinase; MW, molecular weight.

Subsequent to exposure to 1.0 μmol/l imatinib for 48 h, *BCR-ABL* protein levels and PTK activity were assessed in the CD34^+^ cells from patients with CML in the megakaryocytic and myeloid crisis phases. Fig. 5 shows that exposure to 1.0 μmol/l imatinib for 48 h did not reduce the *BCR-ABL* protein levels, but did reduce the PTK activity in the CD34^+^ cells from patients with CML in the megakaryocytic and myeloid crisis phases. However, the decline in PTK activity was not notably different between the CD34^+^ cells from patients with CML in the megakaryocytic crisis phase and those from patients with CML in the myeloid crisis phase.

## Discussion

CML is a disorder characterized by the clonal expansion of BM stem cells, with the characteristic t(9;22)(q34;q11) cytogenetic abnormality that results in Philadelphia chromosome and the generation of a *BCR-ABL* chimeric gene (18). There is no single standard therapy for patients with CML at an advanced stage. The results of a phase II study on CML have indicated that imatinib is a valuable treatment alternative in patients with CML in myeloid blast crisis (10). Megakaryocytic blast crisis as the presenting manifestation of CML is rare. Previous studies (11,12,19), with the exception of a study by Westfall *et al* (20), have shown that imatinib has good effects on patients with a megakaryocytic blast crisis of CML. However, the *in vitro* effect of exposure to imatinib on the proliferation or apoptosis of CD34^+^ cells from patients with CML in the megakaryocytic crisis phase has not been described. To the best of our knowledge, the present study is the first to address the effect of imatinib on the CD34^+^ cells of patients with CML in the megakaryocytic crisis phase.

The present data showed that imatinib induced the G_1_ arrest of CD34^+^ cells from patients with CML in the megakaryocytic crisis phase. This indicates that the antiproliferation effect of imatinib may be connected with the role of the drug in interfering with cell cycle progression. It is well known that the phosphorylation of Rb and the dephosphorylation of CDK1 are significant in the transition of the G_1_/S and G_2_/M phases, respectively (21,22). Rb is a negative regulator of cell proliferation and is inactivated by phosphorylation. The present study demonstrated that phosphorylated Rb and CDK1 are significantly downregulated by imatinib, which is consistent with the G_1_/S, but not the G_2_/M arrest caused by imatinib.

The cellular mechanisms of imatinib have been associated with the induction of apoptosis (23). The activation of procaspase-3 following caspase-8 or caspase-9 activation is considered to be crucial in apoptosis (24,25). The present study showed that imatinib-induced apoptosis coincided with the activation of caspase-3 in the CD34^+^ cells from patients with CML in the megakaryocytic crisis phase. The *BCR-ABL* fusion protein forms the molecular basis of CML. The protein induces the inhibition of apoptosis, the deregulation of cell proliferation and the adhesion abnormalities of the marrow stroma. The application of imatinib to treat CML has therefore been considered to be a molecular success in the genomic era of cancer research. The present data show that imatinib inhibits the tyrosine kinase activity of *BCR-ABL,* but that it does not appear to affect the turnover of this protein.

In conclusion, the present study indicated that imatinib significantly inhibited proliferation, induced apoptosis and reduced the tyrosine kinase activity of *BCR-ABL* in CD34^+^ cells from patients with CML in the megakaryocytic crisis phase. This indicates that imatinib may be a potential chemotherapy drug for patients with CML in the megakaryocytic crisis phase. The treatment for the megakaryocytic crisis phase is similar to the general treatment of CML in the myeloid crisis phase. Clinical trials are required to determine whether this *in vitro* activity will translate into a better response and survival rate for patients with CML in the megakaryocytic crisis phase.

## Abbreviations

BC: blast crisis
CML: chronic myeloid leukemia
BM: bone marrow
AMegL: acute megakaryocytic leukemia
BMMNCs: BM mononuclear cells
RIPA: radioimmunoprecipitation assay
